# Assessing the potential impact of vector-borne disease transmission following heavy rainfall events: a mathematical framework

**DOI:** 10.1098/rstb.2018.0272

**Published:** 2019-05-06

**Authors:** G. Chowell, K. Mizumoto, J. M. Banda, S. Poccia, C. Perrings

**Affiliations:** 1 Department of Population Health Sciences, School of Public Health, Georgia State University, Atlanta, GA 30303, USA; 2 Computer Science Department, Georgia State University, Atlanta, GA 30303, USA; 3 Computer Science Department, University of Turin, 10124 Turin, Italy; 4 School of Life Sciences, Arizona State University, Tempe, AZ 85281, USA

**Keywords:** mosquito-borne disease, vector-borne disease, mathematical model, transmission dynamics, heavy rainfall event, climate change

## Abstract

Predicting the impact of natural disasters such as hurricanes on the transmission dynamics of infectious diseases poses significant challenges. In this paper, we put forward a simple modelling framework to investigate the impact of heavy rainfall events (HREs) on mosquito-borne disease transmission in temperate areas of the world such as the southern coastal areas of the USA. In particular, we explore the impact of the timing of HREs relative to the transmission season via analyses that test the sensitivity of HRE-induced epidemics to variation in the effects of rainfall on the dynamics of mosquito breeding capacity, and the intensity and temporal profile of human population displacement patterns. The recent Hurricane Harvey in Texas motivates the simulations reported. Overall, we find that the impact of vector-borne disease transmission is likely to be greater the earlier the HREs occur in the transmission season. Simulations based on data for Hurricane Harvey suggest that the limited impact it had on vector-borne disease transmission was in part because of when it occurred (late August) relative to the local transmission season, and in part because of the mitigating effect of the displacement of people. We also highlight key data gaps related to models of vector-borne disease transmission in the context of natural disasters.

This article is part of the theme issue ‘Modelling infectious disease outbreaks in humans, animals and plants: approaches and important themes’. This issue is linked with the subsequent theme issue ‘Modelling infectious disease outbreaks in humans, animals and plants: epidemic forecasting and control’.

## 1. Introduction

It is now well understood that human-induced global warming is associated with an increasing risk of extreme weather events [1]. Higher air temperatures have two main effects on extreme weather events. Since warmer air contains more water, extreme weather events increasingly involve high rain rates. At the same time, higher air temperatures have led to atmospheric circulation changes that include a decline in the translation speed of storms—by 10% over the period 1940–2016 [2]. Together, these two effects increase the frequency and intensity of heavy rainfall events (HREs). Although we expect the flooding associated with HREs to have consequences for disease, the short- and long-term effects of these events on the risk of infectious disease epidemics driven by insect distribution patterns remain understudied [3].

In the USA, the frequency of HREs has increased with average temperatures across the country during the past 3–5 decades—especially in the Northeast, Midwest and Great Plains [4]. Several factors make the USA vulnerable to disasters stemming from HREs [5]. The most important of these is the fact that a large and growing population segment (currently about 60 million people) live in coastal cities, many of which are at high risk from hurricanes. Hurricanes Katrina, Sandy, Harvey and Irma all impacted high population density coasts, leaving many without access to basic services like electricity and water. The resulting flooding led to the displacement and death of many individuals.

Extreme weather events are recognized to pose special health hazards [6], including the threat of infectious water-related (e.g. cholera, leptospirosis) [7], soil-transmitted (e.g. helminth infections) [8] and vector-borne infectious diseases (e.g. dengue, chikungunya and Zika) [7]. In Southeastern Texas and South Florida, local climatological conditions promote low-to-moderate abundance of the mosquito *Aedes aegypti* and *Aedes albopictus*—the main vectors for a number of arboviruses including dengue, chikungunya and Zika [9]. Stagnant water left over from HREs leads to increased abundance of mosquitoes in affected regions, while the deterioration of public and private health regimes increases the likelihood that people are infected. In the immediate aftermath of a disaster, individuals within the disaster zone may be at increased risk of infection owing to the breakdown of private and public preventive measures, the disruption of healthcare delivery and increased mosquito densities.

While forecasting the extent and impact of HRE-induced epidemics is challenging, well designed and parametrized mathematical models can be used to simulate the potential trajectory and severity of outbreaks, as well as the impact of control interventions. In this paper, we employ a rainfall-driven mathematical epidemic model to illustrate the potential impact of vector-borne diseases based on the timing of the HREs (e.g. hurricanes) relative to the transmission season, short-term dynamics of mosquito breeding capacity in response to rainfall and the mitigating effect of population displacement. The recent Hurricane Harvey in Texas motivates our transmission scenarios. We highlight key data gaps related to models of vector-borne disease transmission in the context of natural disasters.

## 2. The heavy rainfall event-induced epidemic model

The model allows us to explore the potential impact of HREs on vector-borne disease spread by incorporating key ingredients of vector-borne disease transmission, human displacement patterns, interventions, dynamic mosquito carrying capacity in response to rainfall, case importation rates and the timing of HREs relative to the transmission season. A key element in a model of HRE-induced infectious disease transmission is the change in population mobility and displacement during and after the event [10]. Here, we model the temporal profile of the human population in an affected area, *i*, as a function of the baseline population, denoted $N_{h_{i}}(t)$, and the proportion of the population displaced out of area *i* as a result of the HRE, denoted $H_{i}(t)$. Population displacement affects mosquito-borne epidemics in different ways. First, displacement means that routine habitat control (e.g. emptying water containers) is neglected, which increases the mosquito carrying capacity of the local environment, and hence the risk of being bitten. Second, by reducing the size of the human population, displacement reduces both the number of local infections and the number of external case importations. The rate of external case importations into the area in the absence of an HRE is denoted $\alpha_{i}$, and with an HRE as the product $\alpha_{i}(1-H_{i}(t))$. Although the external importation rate $\alpha_{i}$ would be expected to vary over the year, we take it to be constant over the interval of the event. As a first approximation, we also assume that it is the same across the area impacted by the HRE.

Population mixing patterns vary depending on the severity, spatial extent and duration of the event, as well as on behaviour changes prompted by the evolving characteristics of the event and any evacuation orders. In the immediate aftermath of a disaster, individuals within the disaster zone may be at increased risk of infection owing to the breakdown of private and public preventive measures, the disruption of healthcare delivery and increased mosquito densities.

### (a) Short-term dynamics of mosquito breeding capacity

A number of studies have found a significant link between local climatological factors and the risk of vector-borne disease outbreaks (e.g. [11,12]). For example, an increase in dengue outbreak risk has been associated with increasing minimum temperatures (e.g. [13,14]) and excess rainfall occurring one to two months earlier [13]. Following an HRE, initial flooding and high winds may negatively affect existing mosquito breeding sites [7]. However, as the surface runoff and flooding recede, the number of water-holding containers increases, which directly amplifies mosquito breeding capacity. After the storm, laid eggs hatch, larvae mature and pupae develop into adult mosquitoes (approx. two weeks later). Following a case importation, new cases of the disease may then occur after a generation interval of the disease of about two to three weeks [15]. While a number of studies have shed light on the effects of temperature on the development, survival, reproduction and disease-transmitting capacity of mosquitoes [11], the complex mechanisms through which temperature and rainfall affect the risk of mosquito-borne epidemic outbreaks remain poorly studied. Here, the rainfall-dependent mosquito carrying capacity in location *i* is given by $K_{i}(t)$ and bounded by a maximum mosquito–host ratio denoted by $m_{\max}$. The corresponding rate of change equation is given by

$$\frac{dK_{i}(t)}{dt}=\{\begin{array}{cc} c_{1}N_{h_{i}}J_{i}(t-\tau )-c_{2}(1-H_{i}(t))K_{i}(t) & K_{i}(t)<m_{\max}N_{h_{i}}\\ -c_{2}(1-H_{i}(t))K_{i}(t)\; & \mathrm{otherwise}\; \end{array},$$

where $N_{h_{i}}$denotes the human population residing in area *i* and $J_{i}(t)$ denotes the time-dependent rainfall in location *i*. Because the effects of rainfall on mosquito breeding capacity are not instantaneous but depend on how quickly surface runoff and flooding recede [7], the parameter τ models the delayed impact of rainfall on the generation of new mosquito breeding grounds. Further, the parameter $c_{1}$ quantifies the per capita rate of production of new breeding sites from rainfall, whereas $c_{2}(1-H_{i}(t))$ quantifies the rate at which mosquito breeding sites are destroyed (e.g. emptying water containers around the household), which depends on the proportion of the displaced population in a given area *i*.

### (b) Vector-borne infectious disease transmission dynamics

**Figure 1.**
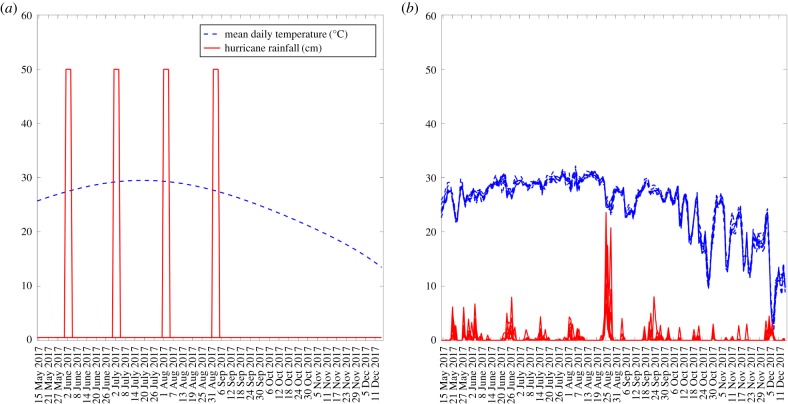
(*a*) Daily temperature and rainfall curves employed for assessing the impact of the timing of the HRE on the epidemic attack rate. We assumed four different 4-day North Atlantic hurricanes characterized by sustained rainfall at 50 cm per day occurring on 1 June, 1 July, 1 August or 1 September and a baseline (nonhurricane) rainfall per day at 0.5 cm, together with a temperature cycle that is consistent with that of the evacuation counties in Texas during Hurricane Harvey. (*b*) Daily temperature and rainfall time series for each of the mandatory evacuation counties in Texas (Arkansas, Brazoria, Calhoun, Jackson, Matagorda, Refugio, San Patricio and Victoria) used for Hurricane Harvey simulation scenarios.

We expanded the baseline compartmental SEIR-type model of arboviral transmission dynamics introduced by Huber *et al*. [16]. In this model, the authors linked the effects of temperature to mosquito reproduction, development, survival and transmission capacity [11]. Local temperature strongly modulates the reproduction, development and disease-transmitting capacity of the mosquitoes. While temperature-dependent risk does not fluctuate substantially in the Tropics where temperature cycles are weak, in temperate areas of the world, including the southern coastal areas of the USA, with well-defined temperature cycles and an Atlantic hurricane season running from 1 June to 30 November, mosquito-borne disease transmission is expected to depend on the timing of the HRE. For example, in the context of a well-defined temperature cycle (figure 1), we can expect a higher epidemic risk following a hurricane that occurs near the peak temperature cycle; whereas a lower epidemic risk may be expected for hurricanes that make landfall near the end of the hurricane season. For illustration, figure 1*a* shows hypothetical scenarios for six different 4-day North Atlantic hurricanes characterized by sustained rainfall at 50 cm d^−1^ relative to a seasonal temperature cycle, which is consistent with that of southeast Texas.

We adapted the Huber model in several ways.

First, the population was divided into spatial areas (e.g. counties) to account for spatial heterogeneities in human population size ($N_{h_{i}}(t)$), mosquito population size ($N_{v_{i}}$(*t*)), temperature ($T_{i}(t)$), precipitation ($J_{i}(t)$), profile of population displacement relative to the HRE ($H_{i}(t)$) and external disease importation rates ($\alpha_{i}$), but we assumed this parameter constant across areas in our simulations. Moreover, the human population in a given area *i* was classified into four epidemiological states: susceptible ($S_{h_{i}}(t)$), exposed ($E_{h_{i}}(t)$), infectious ($I_{h_{i}}(t)$) and recovered ($R_{h_{i}}(t)$), with the cumulative number of infectious humans given by $C_{h_{i}}(t)$, while the mosquito population is classified into three states: susceptible ($S_{v_{i}}(t)$), exposed ($E_{v_{i}}(t)$) and infectious ($I_{v_{i}}(t)$).

Second, local susceptible mosquitoes were assumed to be infected from local infectious humans in area *i* and to a lesser extent from the influence of infectious humans visiting from other areas. For simplicity, we assumed that the rate of transmission from area *j* into area *i* decays exponentially with the Euclidean distance between their respective county centroids denoted by $d_{ij}$. Hence, the spatial contact matrix was scaled by $e^{-qd_{ij}}$, where parameter *q* quantifies the extent of local spatial transmission. That is, small values of *q* lead to broad spatial transmission influence, whereas large values of *q* emphasize local spread. More elaborate forms of the contact matrix are discussed in [17].

Third, the dynamic adult mosquito carrying capacity, $K_{i}(t)$, was taken to respond to the rainfall dynamics as described earlier.

Fourth, because a displaced population resulting from the HRE affects the local mosquito reproduction rate, we also scaled the number of eggs laid per female per day ($\mathrm{EFD}(T_{i})$) and the local force of infection for mosquitoes by the proportion of the population remaining in area *i*, which is given by ($1-H_{i}(t)$).

Finally, we accounted for an external disease importation rate given by $\alpha_{i}(1-H_{i}(t)),$ where $\alpha_{i}$ is the baseline disease importation rate in the absence of an HRE.

The temperature-dependent functional responses of *A. aegypti* and *A. albopictus* and dengue transmission traits are driven by empirical data, which were directly informed by prior work (table 1 in [16]). Briefly, these parameters are as follows: the biting rate ($a(T_{i})$), the number of eggs laid per female per day ($\mathrm{EFD}(T_{i})$), the probability of mosquito-egg-to-adult survival ($p\mathrm{EA}(T_{i})$), the mosquito-egg-to-adult development rate ($\mathrm{MDR}(T_{i})$), the adult mosquito lifespan ($1/\mu (T_{i})$), the probability of mosquito infectiousness ($b(T_{i})$), the probability of mosquito infection ($p\mathrm{MI}(T_{i})$) and the virus extrinsic incubation rate ($\mathrm{PDR}(T_{i})$). Further, the average intrinsic incubation period and the average host infectious period are denoted by 1/*κ* and 1/*γ*, respectively.

The full spatial model of vector-borne disease transmission dynamics is given by the following system of ordinary differential equations (electronic supplementary material, figure S1)

$$\begin{array}{rl} \frac{dS_{v_{i}}}{dt} & =(1-H_{i}(t))\mathrm{EFD}(T_{i})p\mathrm{EA}(T_{i})\mathrm{MDR}(T_{i})\mu {\left(T_{i}\right)}^{-1}N_{v_{i}}\\ & \;\times \log\left(\frac{K_{i}(t)}{N_{v_{i}}}\right)-a(T_{i})p\mathrm{MI}(T_{i})S_{v_{i}}(1-H_{i}(t))\\ & \;\times \left(\sum_{\;j=1}^{n}e^{-qd_{ij}}\frac{I_{h_{j}}}{N_{h_{i}}}\right)-\mu (T_{i})S_{v_{i}},\\ \frac{dE_{v_{i}}}{dt} & =a(T_{i})p\mathrm{MI}(T_{i})S_{v_{i}}(1-H_{i}(t))\\ & \;\times \left(\sum_{\;j=1}^{n}e^{-qd_{ij}}\frac{I_{h_{j}}}{N_{h_{i}}}\right)-(\mathrm{PDR}(T_{i})+\mu (T_{i}))E_{v_{i}},\\ \frac{dI_{v_{i}}}{dt} & =\mathrm{PDR}(T_{i})E_{v_{i}}-\mu (T_{i})E_{v_{i}},\\ \frac{dS_{h_{i}}}{dt} & =-(1-H_{i}(t))\left(S_{h_{i}}a(T_{i})b(T_{i})\frac{I_{v_{i}}}{N_{h_{i}}}+\alpha_{i}\right),\\ \frac{dE_{h_{i}}}{dt} & =(1-H_{i}(t))\left(S_{h_{i}}a(T_{i})b(T_{i})\frac{I_{v_{i}}}{N_{h_{i}}}\right)-kE_{h_{i}},\\ \frac{dI_{h_{i}}}{dt} & =kE_{h_{i}}+(1-H_{i}(t))\alpha_{i}-\gamma I_{h_{i}},\\ \frac{dR_{h_{i}}}{dt} & =\gamma I_{h_{i}}\\ \mathrm{and}\;\frac{dC_{h_{i}}}{dt} & =kE_{h_{i}}+(1-H_{i}(t))\alpha_{i}. \end{array}$$

### (c) Mobility analysis for Hurricane Harvey based on tweet data

A critical component of the adapted model is the proportion of the population displaced by the disaster. There are no direct measures of displacement. Indirect measures include the proportion of the population in evacuation zones together with the existence of evacuation orders, or observations of the number of houses damaged or destroyed. While the first of these could be used to estimate an upper bound on displacement, it is less useful when evacuation is optional or when there is only partial compliance with mandatory evacuation orders. Similarly, while property damage could be used to estimate the number of people forced out of their homes, this is not the same as being forced out of the area. One option that might capture physical displacement is the number of geo-referenced contributions to social media. To calibrate this element of the model, we acquired a large tweet dataset from GNIP [18]. In addition to the text content of the tweets, the dataset provided metadata including the time of the tweet, the ID and the screen name of the user account, as well as location information. Location was provided in the form of point coordinates (specified in latitude and longitude) and/or ‘place’ information encoded in the form of a place name and a bounding box. Per Twitter specifications, the ‘place’ information encoded in the tweet does not necessarily correspond to where the tweet originated from, but may instead represent the spatial context of the content of the tweet, so we dropped all tweets that did not contain precise point coordinates. The data, which were originally formatted as a JSON file, were stored and indexed in MongoDB and queried using Python. To identify tweets originating from a given county, we used the county-level bounding boxes.

For the mobility analysis, we used R [19] and the following packages: Tigris [20], Leaflet [21] and Raster [22]. In order to analyse the mobility of people tweeting within the mandatory evacuation counties, we analysed all tweets (26 million) generated during the period 1 January 2017 to 15 October 2017 from users geo-located in the following states: Texas, Oklahoma, Alabama, Mississippi, Arkansas, Louisiana, Georgia, South Carolina, North Carolina, Florida and Tennessee. While Twitter users in the dataset have specified that their location is in the previously mentioned states, only 1 985 401 tweets contain the actual geo-location where the tweet was originated. We then focused our study period from 17 July 2017 (one month before the first Harvey-related alert was issued) to 15 October 2017, which includes 16 764 geo-referenced tweets within the mandatory evacuation counties in Texas (Arkansas, Brazoria, Calhoun, Jackson, Matagorda, Refugio, San Patricio and Victoria) (electronic supplementary material, figure S2) [23].

To estimate the number of Twitter users who mobilized out of the evacuated counties, we first identified users who lived in those counties based on their tweeting activity during the pre-hurricane period: 17 July 2017 to 20 August 2017. We restricted each user to have at least two tweets within those counties to adjudicate their place of residence [24]. We then analysed their tweeting activity during the hurricane period: 21 August 2017 to 3 September 2017 (last day of hurricane warnings [25]) in order to analyse any tweeting activity within the evacuation counties and in any of the USA states included in our database. Finally, based on tweeting activity, we also estimated the number of users who had returned to their residence in the evacuation counties by 15 October 2017 (end of the dataset).

**Figure 2.**
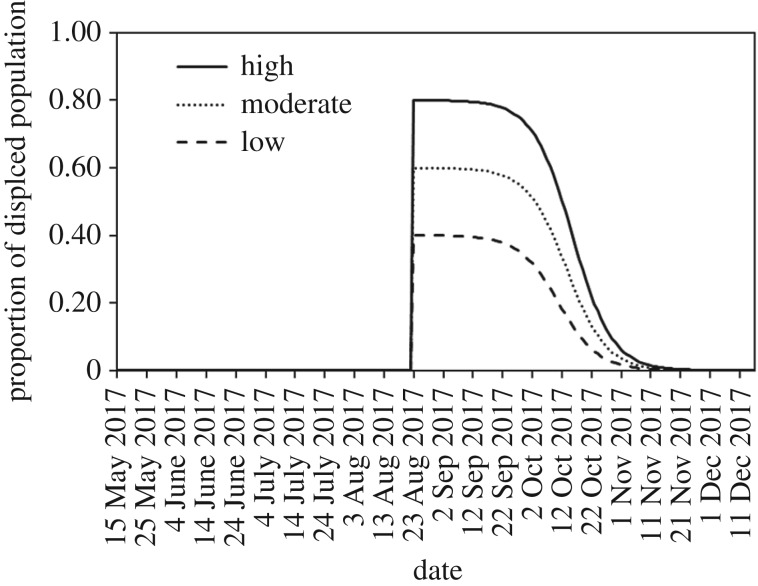
Three response curves (low, moderate, high) for $H_{i}(t)$ describing the proportion of the displaced population in a given area *i* relative to the timing and duration of the HRE that were modelled using two logistic functions: (i) the proportion of the displaced population rapidly starts to increase on 21 August 2017 until a maximum displacement level $H_{\max}$ is reached 1 day later and (ii) the proportion displaced gradually declines from $H_{\max}$ until baseline pre-disaster levels return 100 days later. The values of $H_{\max}$ were informed by our tweet-based analyses suggesting that the proportion of the displaced population during Hurricane Harvey ranged from 38 to 80%.

We chose to model $H_{i}(t)$, describing the proportion of the displaced population in a given area *i* relative to the timing and duration of the HRE, using two logistic functions: (i) the proportion of the displaced population rapidly starts to increase on 21 August 2017 until a maximum displacement level $H_{\max}$ is reached 1 day later and (ii) the proportion displaced gradually declines from $H_{\max}$ until baseline pre-disaster levels return 100 days later. The values of $H_{\max}$ were informed by our tweet-based analyses (figure 2).

### (d) Baseline parameter values and initial conditions

Simulations start on 15 May 2017 and end on 15 December 2017. The initial mosquito carrying capacity in an area *i* prior to the HRE event (parameter $K_{b_{i}}$) is given by the product of the pre-HRE ratio of mosquitos per person (denoted by m) and the human population size, $N_{h_{i}}$. Further, the initial adult mosquito population was assumed to be at carrying capacity and entirely susceptible. Because we focus on short-term epidemic dynamics following HREs, we assume a constant and initially completely susceptible host population before five initial infectious humans are introduced in the county of Aransas, Texas. This is consistent with the fact that only small autochthonous outbreaks of dengue and Zika have been documented in the region [26]. The average intrinsic incubation period (1/k) and the average host infectious period (1/γ) were fixed at 5.9 days and 5 days, respectively, as in [16]. Other model parameter values and their uncertainty ranges are given in table 1.

**Table 1.** Baseline and uncertainty ranges for model parameters.

parameter	definition	baseline value	range
α	average case importation rate (1/day)	1/14	0–1/7
$c_{1}$	per capita rate or production of new breeding sites from rainfall (1/(person × rainfall))	0.02	0.01–0.2
$c_{2}$	rate at which people destroy mosquito breeding sites (1/day)	0.02	0.01–0.2
τ	delayed impact of rainfall on the generation of new mosquito breeding grounds (days)	14	14–21
m	initial mosquito–host ratio prior to the HRE	0.2	0.1–1
$m_{\max}$	maximum mosquito–host ratio	8	6–10
*q*	parameter quantifying the extent of local transmission (1/km)	0.0001	0.01–0.00001

### (e) Simulations for model testing, verification and assessing the impact of the timing of the heavy rainfall event on the epidemic attack rate

We simulated outbreaks for four different hypothetical 4-day hurricane scenarios characterized by sustained rainfall at 50 cm d^−1^ occurring on 1 June, 1 July, 1 August or 1 September and a baseline (non-hurricane) rainfall per day at 0.5 cm, together with a temperature cycle that is consistent with that of the evacuation counties in Texas during Hurricane Harvey (figure 1*a*). For these simulations, we modelled a single population of 100 000 people.

Baseline simulations of no-HREs were obtained by assuming a constant rainfall level at 0.5 cm d^−1^ and assuming no population displacement (i.e. $H_{i}(t)=0\;\;\mathrm{for}\;\;\mathrm{all}\;\;i$).

### (f) Simulations specifically tailored for Hurricane Harvey in Texas

The model was parametrized on data from Hurricane Harvey, Texas, 2017. This affected the Greater Houston Area in Southeast Texas with a population of around 2.3 million [27]. The population includes many people of low socio-economic status, known to be at high risk of arboviral diseases (e.g. West Nile Virus, dengue, chikungunya and Zika) transmitted by *A. aegypti* and *A. albopictus* mosquitoes [27,28]. The hurricane, the most severe extreme rainfall event in USA history, crossed the coast of Texas on 24 August 2017 as a category-4 hurricane, bringing torrential rain of over 127 cm on parts of the greater Houston area over the course of 4 days, and leading to flood damage estimated at $125 billion [25]. We focus our study on the geographical area in Texas comprised by the counties with a mandatory evacuation order: Arkansas, Brazoria, Calhoun, Jackson, Matagorda, Refugio, San Patricio and Victoria [23].

Annual population size estimates in mid-year as well as daily mean temperature and precipitation across counties for 2017 were obtained from United States Census Bureau [29] and the PRISM Climate Group [30], respectively. We retrieved county-level latitude and longitude coordinates [31] to estimate inter-county Euclidean distances. The county-level population size, mean temperature and total precipitation are shown in electronic supplementary material, figure S3. Daily temperature and rainfall time series for the evacuation counties in Texas for Hurricane Harvey simulation scenarios are shown in figure 1*b*.

Baseline simulations of no-HREs were obtained by limiting the daily rainfall level to 4 cm and assuming no population displacement (i.e. $H_{i}(t)=0\;\;\mathrm{for}\;\;\mathrm{all}\;\;i$) while parameter *q* quantifying the extent of local transmission was varied in the range 0.01–0.00001 (electronic supplementary material, figure S4).

### (g) Uncertainty and sensitivity analyses

We conducted uncertainty and sensitivity analyses to assess the effects of six uncertain parameters: α, $c_{1}$, $c_{2}$, τ, *m* and $m_{\max}$ on the total number of cases occurring during our study period (table 1). For this purpose, we generated 1000 samples of the parameters using a uniform Latin hypercube sampling design (parameter ranges given in table 1) and holding other parameters fixed to their baseline values. For each set of parameter values and different timing of the HRE, we simulated incidence curves and recorded the total number of infectious humans during our study period. We ranked the sensitivity of the parameters based on their effect on the cumulative number of cases according to their partial rank correlation coefficients (PRCC) [32]. Model simulations were generated using the ode45 function in Matlab (Mathworks).

## 3. Results

**Figure 3.**
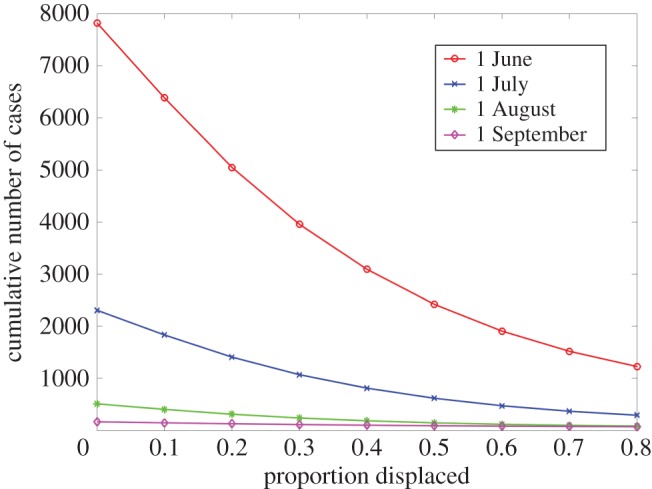
The relation between the timing of an HRE event and the proportion of the population displaced on the cumulative number of cases for the four different hypothetical 4-day hurricane scenarios characterized by sustained rainfall at 50 cm per day occurring on 1 June, 1 July, 1 August or 1 September and a baseline (non-hurricane) rainfall per day at 0.5 cm together with a temperature cycle that is consistent with that of the evacuation counties in Texas during Hurricane Harvey.

We found that the primary drivers of the impact of HREs on mosquito-borne infectious disease include the timing of those events relative to the transmission season, and the proportion of the population displaced during an HRE event. The risk of outbreaks is highest if an HRE occurs early in the transmission season, and lowest if it occurs late in the season. For instance, the cumulative number of cases decreases by 70% for the scenario with an HRE occurring on 1 July relative to the scenario with an HRE occurring on 1 June in the absence of population displacement. Since the net effect of population displacement on disease risk is negative—the more people displaced the lower the risk to those who remain—risk is decreasing in the proportion of the displaced population. The relation between the timing of an HRE event and the proportion of the displaced population is shown in figure 3. Low displacement during events that occur early in the transmission season are associated with the highest number of cases (figure 3).

For the parametrization associated with Hurricane Harvey, we found that our baseline simulations (no-HRE events) did not yield sustained outbreaks. This is consistent with the historic evidence of only small, autochthonous outbreaks of dengue and Zika in Texas [26]. Nor did the addition of an HRE event parameterized on the temperature and rainfall conditions brought by Hurricane Harvey change this. While the event increased the carrying capacity of the local system for *Aedes* species, population displacement reduced the number of imported cases (electronic supplementary material, figure S5). Given temperature and other conditions associated with the timing of the event—Hurricane Harvey crossed the coast of Texas on 24 August 2017—the net effect involved no increase in the risk of local transmission.

**Figure 4.**
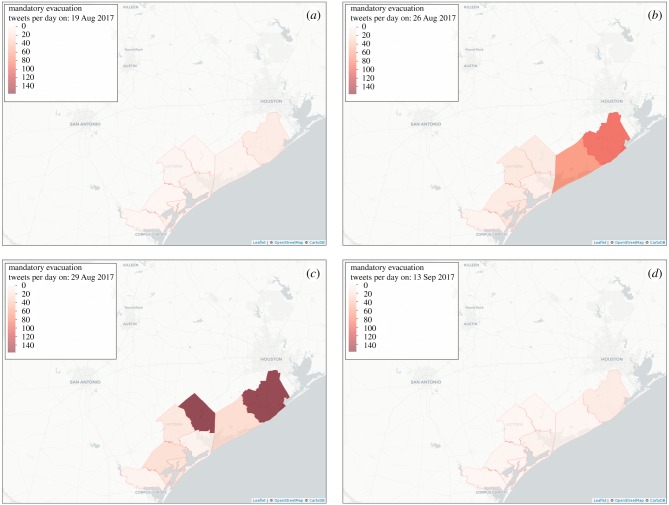
Four different time snapshots of tweeting activity in the mandatory evacuation counties in Texas before (*a*), during (*b*,*c*) and after (*d*) Hurricane Harvey.

**Figure 5.**
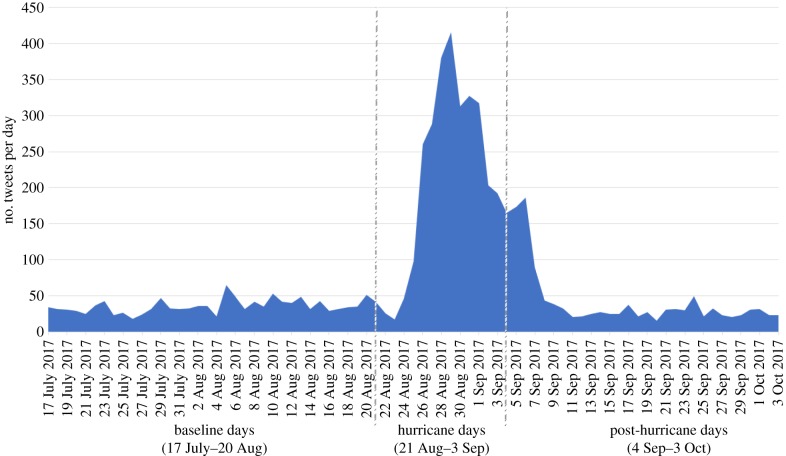
Daily series of the number of tweets before, during and after Hurricane Harvey generated in the mandatory evacuation counties in Texas.

Four different time snapshots of tweeting activity before, during and after Hurricane Harvey are shown in figure 4, while the corresponding total number of tweets is shown in figure 5. From our mobility analysis, we identified 103 unique users living in the evacuation counties during the pre-hurricane period and estimated that 82 of those users had left the evacuation counties as they did not have any tweeting activity within those counties during the hurricane period. Moreover, out of those 82 users, 39 users tweeted at least once outside the evacuation counties. Hence, this suggests that the proportion of the displaced population during Hurricane Harvey ranged from 38% (39/103) to 80% (82/103). In addition, we found that only 12 of the 83 users (12%) had returned to their residence in evacuation counties by 15 October 2017.

To identify the conditions that would have yielded an epidemic, we conducted sensitivity analyses around six parameters (α, $c_{1}$, $c_{2}$, τ, *m* and $m_{\max}$, table 1). We found that a number of these parameters significantly influence the epidemic size (*p* < 0.05), albeit in different ways as shown in electronic supplementary material, figure S6A. Parameters $c_{1}$, $c_{2}$, relating to the creation and removal dynamics of the mosquito carrying capacity, had the most influence on cumulative cases. As expected, the parameter $c_{1}$ has a positive impact on the epidemic size, whereas the parameter $c_{2}$ has a negative impact. The case importation rate (α) also had substantial positive impact on the epidemic size, and the corresponding PRCC increased slightly with later timing of the HRE (electronic supplementary material, figure S6A). Further, the parameter τ negatively influenced epidemic size, which increased slightly with a later timing of the HRE, while the initial vector–host ratio (m) played a more significant role on epidemic size (PRCC = 0.38–0.49) than the maximum vector–host ratio $m_{\max}$ (PRCC = 0.06–0.13). Our findings from sensitivity analyses substantiate a significant decline in the median epidemic size, the later the HRE occurs (electronic supplementary material, figure S6B).

## 4. Discussion

It is widely recognized that natural weather disasters, including HREs, have the potential to increase mosquito-borne disease transmission by changing the availability of breeding sites. Depending on the species of mosquito involved and its breeding site preferences, HREs can have a larger or smaller impact on mosquito abundance. This effect can be amplified by disruption of vector control operations. At the same time, damage to housing (including protective measures such as mosquito screens) and public health infrastructure can increase exposure. The most important risk factors are, however, related to population displacement, and in particular to the conditions in which displaced people find themselves [7]. One of the most extreme examples of the impact of a natural disaster on vector-borne infectious disease is the malaria epidemic that followed the 1991 earthquake in Costa Rica. The April 1991 earthquake was followed by flooding in the August of the same year. The result was a 4700% increase in the incidence of malaria in the worst affected canton over the average monthly rate for the pre-earthquake period. While mosquito habitat changes owing to landslides, river damming and river rerouting were a factor, other important drivers were the disruption of vector control activities and local population displacement that led to increased exposure to mosquitoes [33]. The evidence from flood events elsewhere underlines the importance of the combination of conditions faced by the displaced population. Infectious disease risks depend on an ‘epidemiologic triad’: changes in the conditions of displaced people, changes in the ecosystem of pathogens and changes in the biophysical environment. Risks are highest where displaced people and refugees face overcrowded shelters, poor water and sanitation, poor nutrition and hygiene, and disrupted healthcare [34].

In this paper, we model the potential impact of HREs on mosquito-borne disease transmission in temperate areas of the world such as the southern coastal areas of the USA. We test the disease-risk implications of variation in (i) the intensity and temporal profile of human population displacement away from the area immediately affected by the HRE; and (ii) the effects of rainfall on mosquito abundance. Since human population displacement is not within the area affected by the HRE, and does not have implications for crowding, sanitation, nutrition or hygiene, it is risk-reducing. By contrast, changes in mosquito breeding habitat are risk-increasing, but this is also highly sensitive to when the event occurs in the transmission season. We find that the impact of vector-borne disease transmission is on average greater the earlier an HRE occurs in the transmission season, and the larger the case importation rate.

Our sensitivity analyses underscore the need to improve understanding of the mechanisms connecting HREs and mosquito reproduction and development, and to enhance empirical data on vector control and disease importation after a disaster. The mechanisms connecting HREs and mosquito reproduction concern the link between rainfall and the growth of breeding sites. We have assumed a linear relation between rainfall and the growth of breeding sites. Monitoring the dynamics of flooding at fine spatial–temporal scales is crucial for appropriately modelling the spatial heterogeneity in mosquito breeding capacity as well as for the implementation of preventive and mitigation efforts. Open satellite imagery provides data at coarse spatial scales and is only useful to identify the most heavily affected regions, whereas high-resolution satellite mapping is not available to the public. Rigorous vector monitoring operations, as soon as possible after an emergency, would help improve model projections and hence the capacity to manage HRE-induced epidemics.

Disease importation rates in the model are sensitive to the number of people displaced from the area. They fall with the number of people displaced from the area during an HRE. They rise with the number of people returning after the event, and by the number (and origin) of people moving into a disaster area offering emergency relief, or repair, rehabilitation and restoration work. We do not model the latter, but note that it is potentially extremely important. Short-term changes in mobility patterns that are not explicitly taken into account (see also [10]) might be recovered from data obtained from social media platforms such as Twitter. Such platforms are already important tools for disaster management [35]. Data streams from these sources could be useful to quantify the level of public awareness during emergencies [36] and may provide a useful proxy of the temporal profile of population displacement patterns away from the affected areas.

Social media data do pose several challenges. For instance, to obtain meaningful mobility patterns, existing studies (such as [37]) aggregate geo-coded tweet data for extended periods of time: this ensures that there is a sufficient number of tweets that paint an overall picture of the mobility patterns within the given region. Since only a small portion of all tweets contain point coordinate information, using Twitter data to discover short-term mobility patterns immediately before, during and immediately after a hurricane may be problematic. In addition, there is no evidence that Twitter users are representative of the general population. Indeed, in the USA, Twitter users have been found to overrepresent the more densely populated regions of the country, and to represent a highly non-random sample of the distribution of the general population by gender and ethnicity [38]. Elsewhere, they have been shown to be younger and more educated than the general population [39]. If these characteristics are correlated with mobility, and if evacuations are voluntary, we would expect Twitter data to overestimate the percentage of the population displaced. There is currently no systematic collection of data on the demographics of disaster response. More accurate data on both evacuees and emergency responders would help improve projections of disease risks.

Finally, one limitation of the model developed here is that it is deterministic, capturing the average dynamics of HRE-induced epidemics. Stochastic models would be useful for investigating questions relating to the probability of disease invasion and stochastic extinction [26,40]. Future versions of the model could also be cast as a near real-time forecasting tool to guide the public health interventions based on real-time forecasts of temperature and rainfall during the HRE and scenarios for changes in population mobility and displacement patterns.

## Supplementary Material

Supplementary figures

## Data accessibility

Datasets employed in this study are publicly available except for the Twitter data, as per our data, usage agreement with GNIP, Inc.
