# Supplementary material for: Assessing the potential impact of vector-borne disease transmission following heavy rainfall events: a mathematical framework
Source: Philos Trans R Soc Lond B Biol Sci. 2019 May 6;374(1775):20180272. doi: 10.1098/rstb.2018.0272 (PMC6553605; doi:10.1098/rstb.2018.0272)

**Supplementary Figures**

**Figure S1.**  Schematic diagram of the full spatial model of vector-borne disease transmission dynamics. The model allows us to explore the potential impact of HREs on vector-borne disease spread by incorporating key ingredients of vector-borne disease transmission, human displacement patterns, interventions, dynamic mosquito carrying capacity in response to rainfall, case importation rates, and the timing of HREs relative to the transmission season.

**
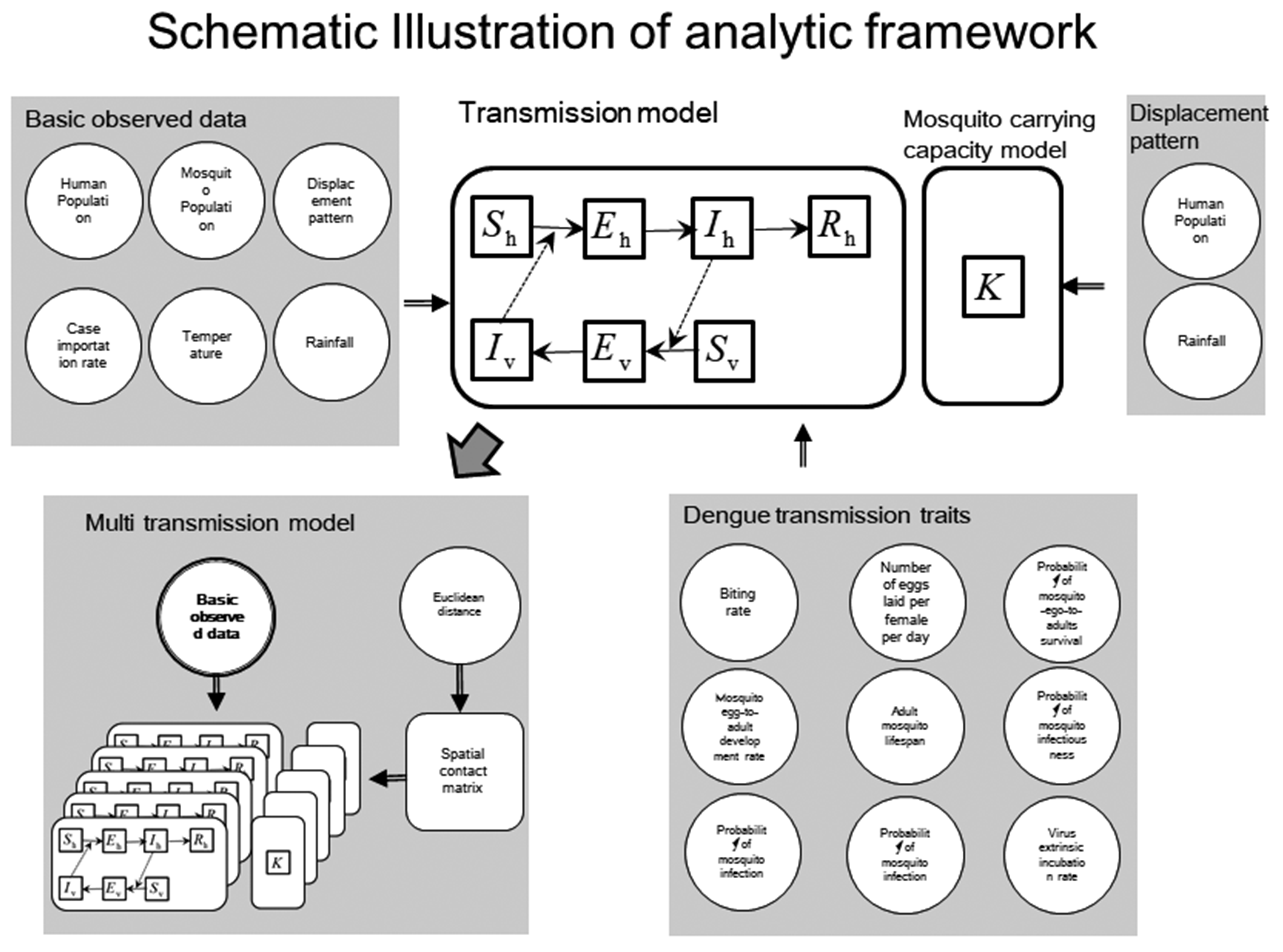
**

**Figure S2**. Map showing the mandatory evacuation counties in Texas: Arkansas, Brazoria, Calhoun, Jackson, Matagorda, Refugio, San Patricio and Victoria.


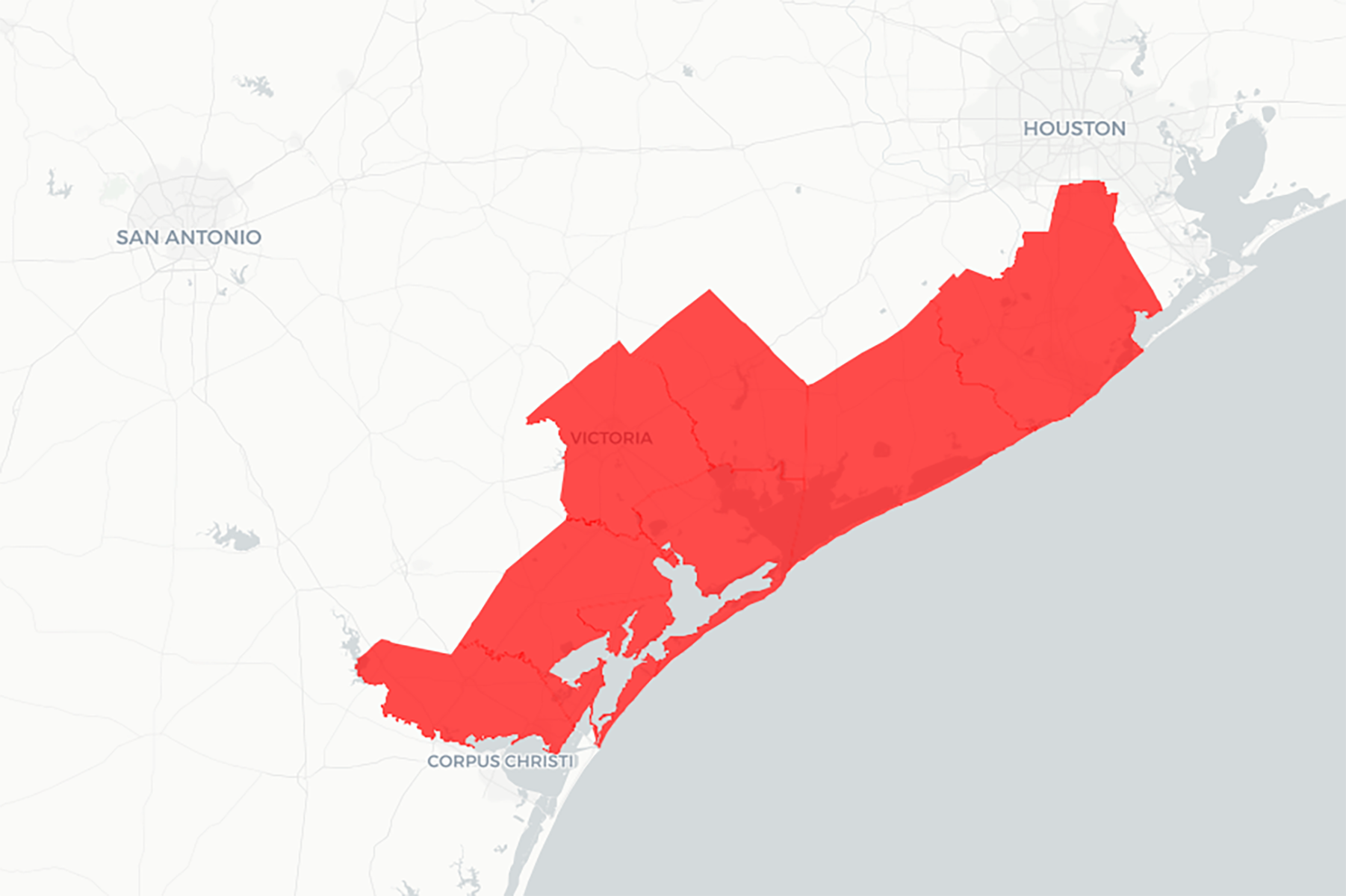


**Figure S3**


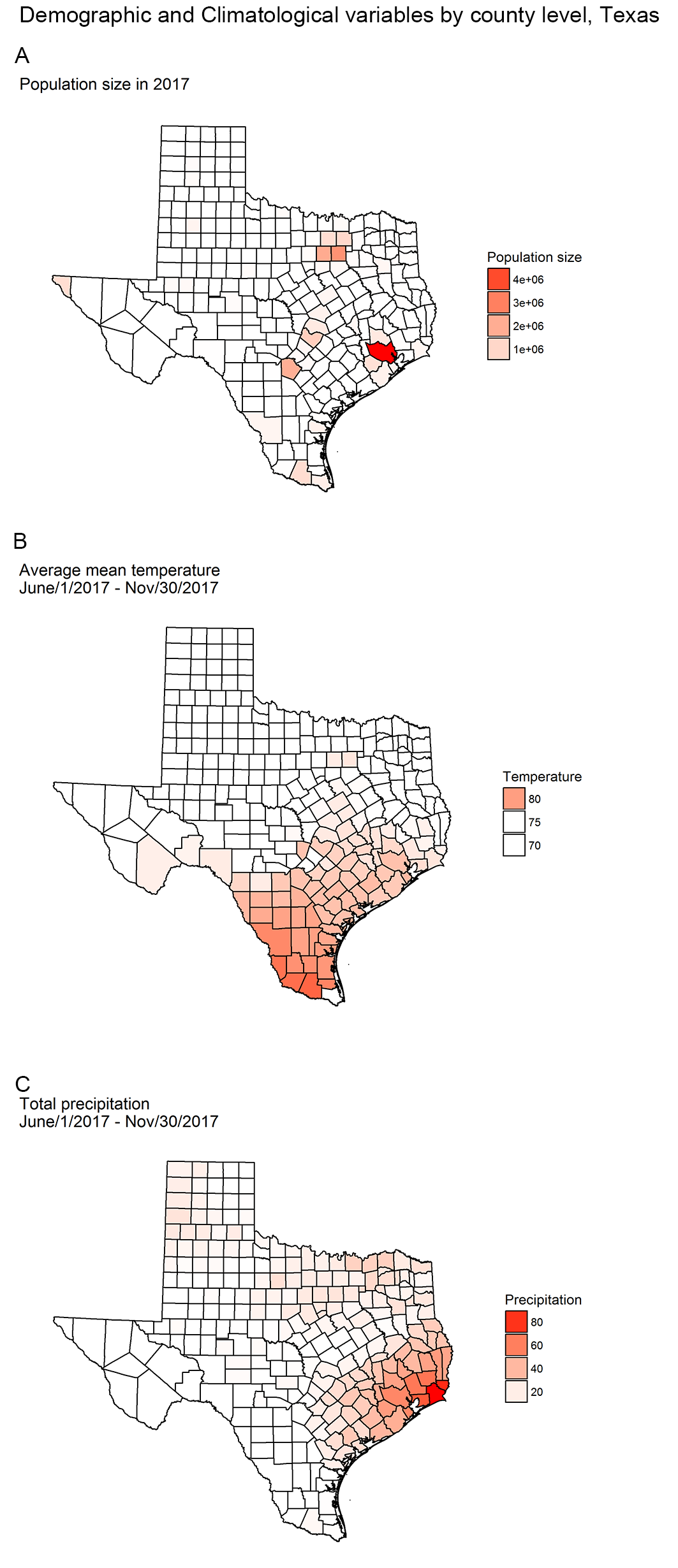


**Figure S4**

Baseline simulations of no-HREs transmission scenarios guided by Hurricane Harvey in the evacuation counties in Texas. For this purpose, daily rainfall level was limited to 4 cm and $H_{i}(t)=0\text{ for all }i$ while parameter $q$ quantifying the extent of local transmission was varied in the range 0.01-0.00001.

**
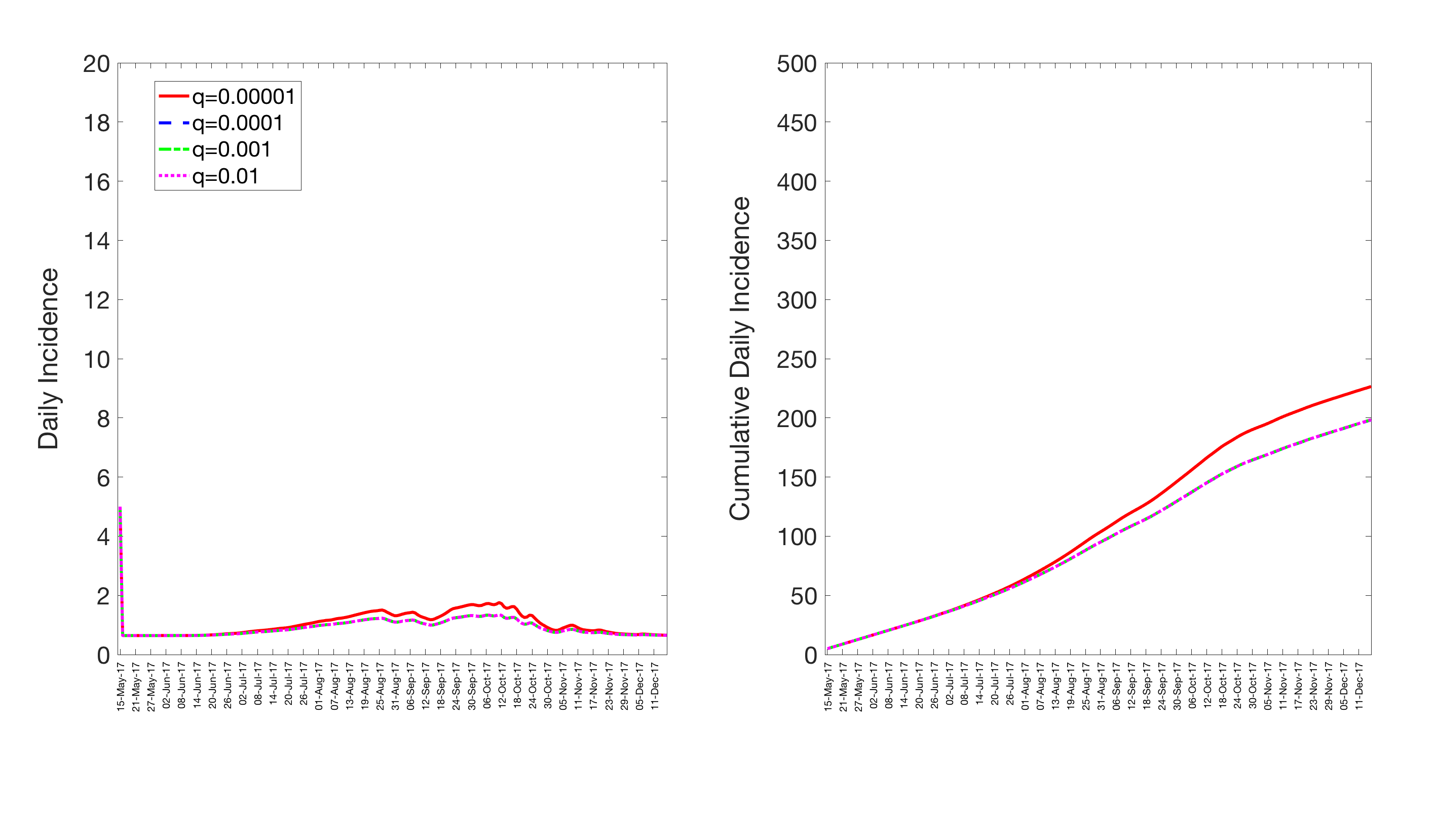
**

**Figure S5**

Simulations for transmission scenarios tailored to Hurricane Harvey in the evacuation counties in Texas using rainfall and temperature data shown in Figure 1B.


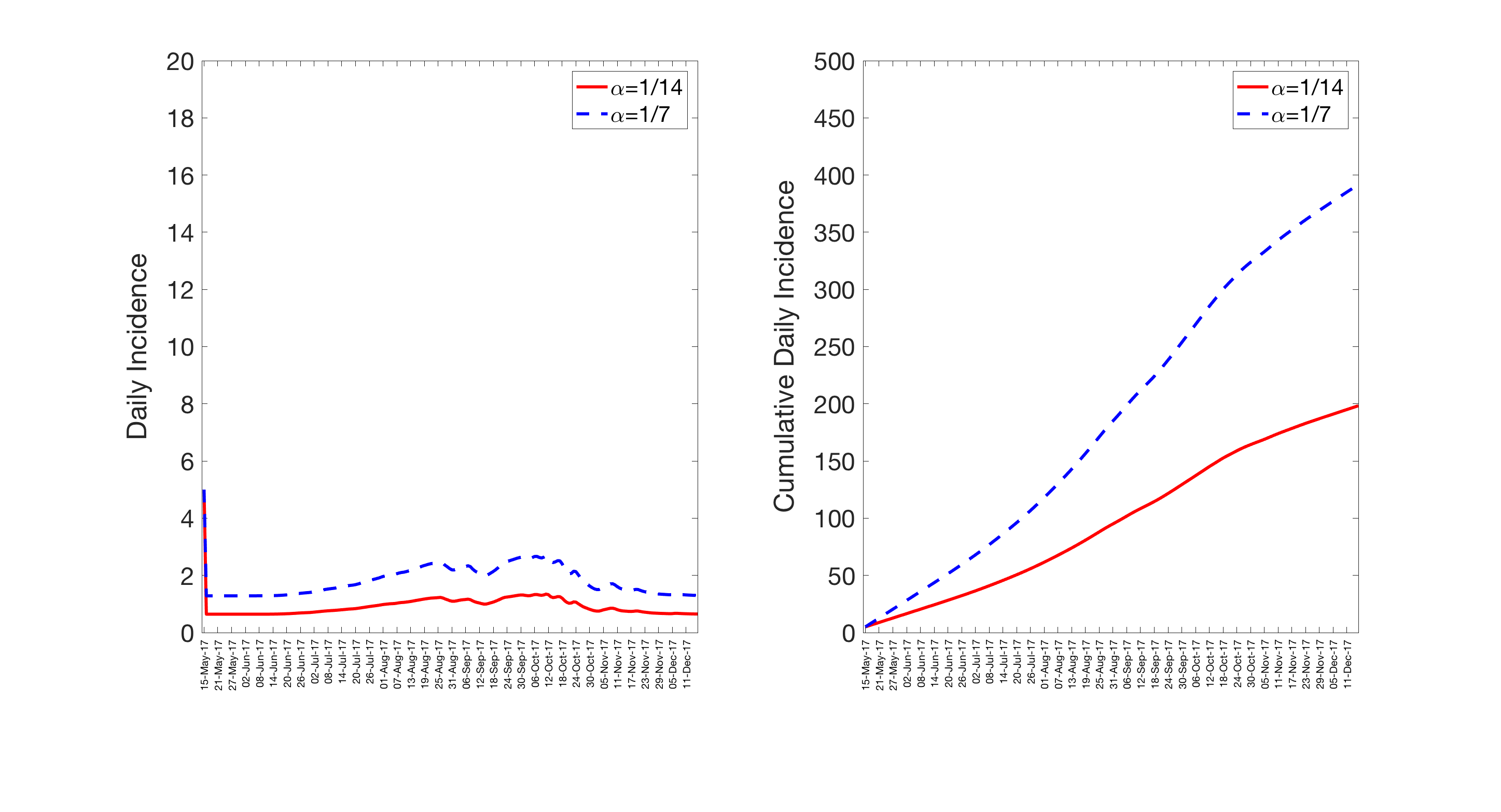


**Figure S6**

A) Partial rank correlation coefficients (PRCC) and B) median epidemic size resulting from our sensitivity analyses on vector-borne disease dynamics based on the four North Atlantic hurricane scenarios with three different response curves (low, moderate, high) for the population displacement displayed in Figure 2.


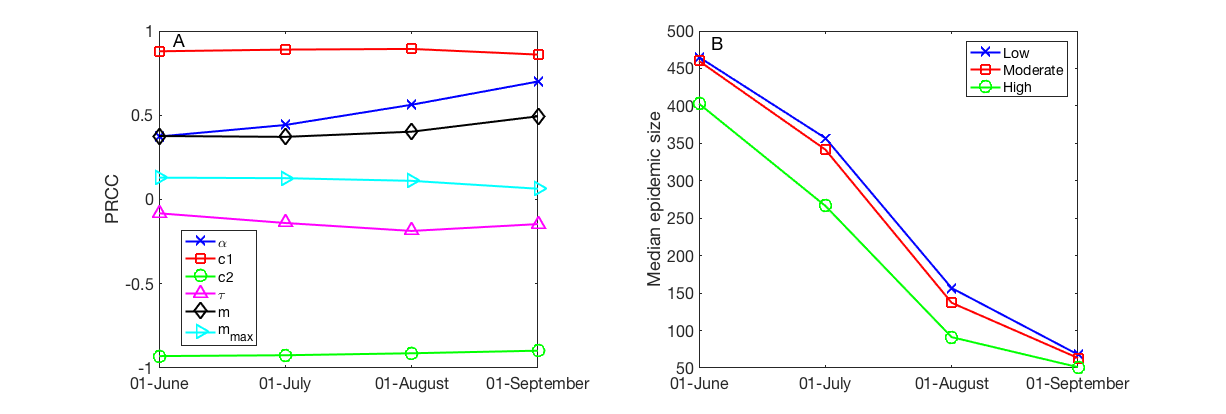

Supplement: Supplementary figures [file rstb20180272supp1.docx]
